# Pressure Mapping Mat for Tele-Home Care Applications

**DOI:** 10.3390/s16030365

**Published:** 2016-03-11

**Authors:** Jose Francisco Saenz-Cogollo, Massimiliano Pau, Beatrice Fraboni, Annalisa Bonfiglio

**Affiliations:** 1 TechOnYou s.r.l, Via La Guardia n.9/A, Villasor (CA) 09034, Italy; 2 Department of Electrical and Electronic Engineering, University of Cagliari, Piazza d’Armi snc, Cagliari 09123, Italy; annalisa@diee.unica.it; 3 Department of Mechanical, Chemical and Materials Engineering, University of Cagliari, Piazza d’Armi snc, Cagliari 09123, Italy; massimiliano.pau@dimcm.unica.it; 4 Department of Physics and Astronomy, University of Bologna, Viale Berti Pichat 6/2, Bologna 40127, Italy; beatrice.fraboni@unibo.it

**Keywords:** pressure sensor, electronic textile, PEDOT:PSS, plantar pressure mapping

## Abstract

In this paper we present the development of a mat-like pressure mapping system based on a single layer textile sensor and intended to be used in home environments for monitoring the physical condition of persons with limited mobility. The sensor is fabricated by embroidering silver-coated yarns on a light cotton fabric and creating pressure-sensitive resistive elements by stamping the conductive polymer poly(3,4-ethylenedioxythiophene):poly(styrene sulfonate) (PEDOT:PSS) at the crossing points of conductive stitches. A battery-operated mat prototype was developed and includes the scanning circuitry and a wireless communication module. A functional description of the system is presented together with a preliminary experimental evaluation of the mat prototype in the extraction of plantar pressure parameters.

## 1. Introduction

The quantification of fundamental data about human posture and movement, such as balance and foot-ground interaction is a fundamental aspect for the evaluation of the quality of life of subjects with limited mobility, such as elderly persons [1]. Most chronic disorders, like arthritis, diabetic foot, and neurodegenerative diseases result in a limitation of mobility and physical activity of the affected persons that, if not controlled on time, can rapidly compromise autonomy [2]. Therefore, technological solutions that allow everyday monitoring of physical function can be used not only to follow the effects of medical conditions and treatments, but also to respond opportunely and prevent or slow down pathologic processes and risk conditions [3].

One of the strategies to assess and monitor the physical condition in a non-intrusive way is by means of the analysis of the patient weight distribution against the floor using force platforms [4]. However, since those instruments are usually bulky and expensive, their use is limited to specialized clinics and research labs and, therefore, cannot be used for long-term daily monitoring. Another possibility is the use of insole systems for monitoring plantar pressure during daily life activities [5]. However, such an approach is usually limited by the low spatial resolution [6] and implies wearing a device that may be difficult to wear or uncomfortable [7].

Here we propose a new mat-like textile pressure mapping device that offers a reasonable trade-off between high-resolution, high-accuracy, but bulky and expensive, force platforms, and wearable, but low-resolution, insole systems. Our device is based on a pressure sensor made of a single layer of a 100% cotton fabric embroidered with silver coated polyester yarns and treated at specific spots with polystyrene sulfonate-doped poly-3,4-ethylenedioxythiophene (PEDOT:PSS). This results in a very thin and flexible textile device that can be easily rolled up during transport and storing and, thus, it can be quickly installed and removed from a home environment.

A similar solution may be developed with plastic film-based pressure sensors [8,9], but a fiber-based alternative can offer superior comfort, flexibility, and mechanical robustness. Those properties allow being better adapted not only to frequent roll and unroll operations, but also to the integration with other textiles in the home environment (carpets, furniture covers, *etc.*) and garments.

The idea of using textile technology for developing large area pressure sensors is not new [10], but until now textile sensors either imply the use of multilayered configurations of fabrics and films that limit the flexibility and make intensive use of expensive materials [11,12,13] or require the fabrication of specially-structured fibers [14,15,16]. In contrast, the proposed sensor is based on conventional cotton fabric, allows using a relatively low amount of conductive polymers and metal-coated yarns, and relies on a fabrication process that deals with only one layer of fabric. Our sensor actually requires including two additional cover layers for providing moisture and mechanical protection, but such layers can be made of conventional low-cost fabrics and do not participate in the transduction process.

In this work, we present the development of a pressure sensitive mat prototype intended to be used in home environments for monitoring the physical condition of elderly persons by means of plantar pressure measurements. In particular, the mat is designed to obtain data about possible anomalies in foot-ground contact (which are typical of metabolic diseases like diabetes or musculoskeletal alteration, such as hallux valgus, toe deformities, flat foot, *etc.* [17]) or assess static and dynamic balance abilities of an individual by analyzing center of pressure (COP) trajectories during static standing or sit-to-standing tasks.

The device is composed of 1024 sensing elements (*i.e.*, sensels) arranged in a 32 × 32 array and covering an area of 39 cm × 39 cm which is enough to accommodate an average person standing with their feet shoulder breadth apart. The prototype includes multiplexing electronics for measuring pressure at high speed; programmable polarization and amplification circuits in order to compensate sensel-to-sensel variability; and modules for wireless LAN communication and web-based user interfaces in order to allow using it locally or remotelly through any LAN-connected device (e.g., SmartTV, tablet, *etc.*). A functional description of the sensor is presented together with a preliminary validation of the mat prototype for extracting plantar pressure parameters.

## 2. Materials and Methods

### 2.1. Fabrication of the Single-Layer Textile Pressure Sensor

**Figure 1:**
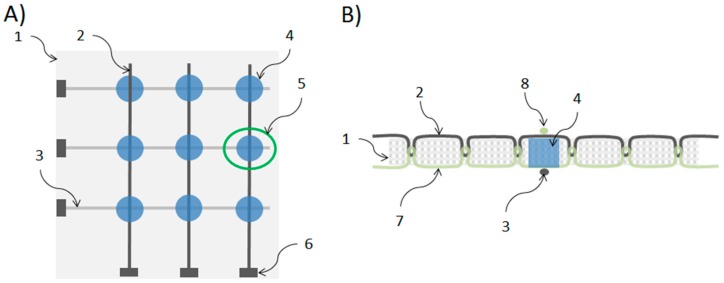
Scheme of a textile pressure sensor. (**A**) Top view of a 3 × 3 array where it is possible to identify: (1) base fabric, (2) top conductive path, (3) bottom conductive path, (4) conductive polymer treated zone, (5) single sensing element or sensel, and (6) connector or electrical contact for measuring; (**B**) Transversal view of a sensel (5 of panel A) illustrating the relation between the base fabric (1), the conductive threads (2 and 3), the non-conductive threads (7 and 8) and polymer-treated zone (4) that create the force-sensitive structure (the connector (6) is not shown in this view).

A single force-sensing element (**5**) consists of a piece of base fabric (**1**) treated with a conductive polymer solution at a crossing point (**4**) between top (**2**) and bottom (**3**) conductive threads that form a force-sensitive electrical connection with the treated fabric (see Figure 1). The base fabric used is a standard 100% cotton fabric commonly used for light bedclothes. Conductive threads are commercial silver coated yarns commonly used in textile electronics [11]. The treatment is done with an aqueous dispersion of PEDOT:PSS mixed with a variable percentage of ethylene glycol which acts as the second dopant [18]. The surface resistivity of the fabric after treatment is usually 10 kΩ/sq. The treatment was done manually using a small cotton stamp for printing the polymeric solution on the spots of interest.

The fabrication procedure starts by sewing the silver-coated yarns directly on the cotton fabric with stitches of the type ISO# 301 (Lockstitch) providing parallel conductive paths on both sides of the fabric as illustrated on Figure 1. Conductive paths on one side of the fabric are orthogonal to, and electrically isolated from, the conductive paths on the opposite side. Since each stitching line is made by two threads (needle and bobbin threads), top conductive paths (**2**) are obtained when using the silver-coated yarn as the needle thread and non-conductive (100% cotton) yarns as the bobbin threads. Conversely, bottom conductive paths (**3**) are obtained when using the silver-coated yarn as bobbin thread and non-conductive (100% cotton) yarn as needle threads. In order to avoid short circuits between top and bottom conductive paths, single stitiches are carefully distributed and arranged in such a way that interlacings belonging to the top paths never become in contact with the interlacings of the bottom paths. The treatment of the crossing points (**4**) is made after the sewing process.

### 2.2. Pressure Sensing Model

The resistance Rm measured at the ends of two orthogonal sewing paths (assuming all other paths driven to ground) can be expressed as:

(1) $$Rm=Rf_{top}+Rs+Rf_{bottom}$$

where $Rf_{top}$ and $Rf_{bottom}$ are the linear resistance of the top and bottom conductive threads, respectively, and Rs is the resistance of the sensel which in turn can be expressed as:

(2) $$Rs=Rc_{top}+Rt+Rc_{bottom}$$

where Rt is the transverse resistance of the treated fabric and $Rc_{top}$ and $Rc_{bottom}$ are the contact resistances between the fabric and top and bottom threads, respectively. Those resistances depend both on the resistivity of threads and fabric fibers, and the force that presses and produces the contact between them. A precise mathematical description of the behavior of those resistances can be quite complex [19]. For the sake of simplicity, however, we can assume that the dominant physical phenomenon is the contact of multiple conductive surfaces and that the overall behavior of the sensel resistance Rs can be modeled as:

(3) $$Rs=kF^{-n}$$

where k is a constant that depends on the geometric conditions and the resistivity of the materials and F is the equivalent force due to the pressure applied on the surfaces. The value of n depends on the nature of the contact and the elasticity of the materials, but it is close to 1 for models where the load causes an increase of the size and number of contact points [20]. Since F is proportional to the applied pressure P, and the resistances $Rf_{top}$ and $Rf_{bottom}$ can be assumed constants during a measure, Equation (1) can be rewritten as:

(4) $$Rm=\frac{a}{P}+b$$

with a and b constants.

### 2.3. Data Acquisition Electronics

**Figure 2:**
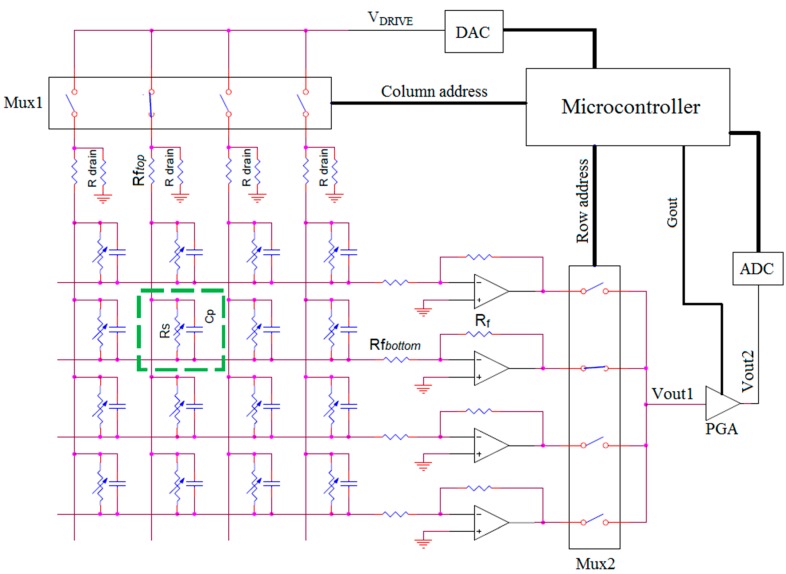
Simplified scheme of the data acquisition electronics.

In order to acquire the signals from the pressure sensors, we developed multiplexing electronics based on the work previously reported by Papakostas *et al.* [9] (a similar approach was also described in detail by Shimojo *et al.* [21]). Briefly, as schematized in Figure 2, each sensel is represented by its resistance Rs in parallel with a parasitic capacitance Cp, and the linear resistances of top and bottom yarns represented by $Rf_{top}$ and $Rf_{bottom}$ respectively. Top and bottom conductive paths on the fabric are respectively associated to column and row connections on the electronics. The management of analog and digital signals is done with a microcontroller which implements the logic for connecting, sequentially, each column to a voltage $V_{DRIVE}$ by means of the multiplexer *Mux1* and each row to the output Vout1 by means of multiplexer *Mux2*. Non-active columns remain referred to the ground by means of the resistances Rdrain in order to reduce the effect of Cp. The use of transimpedance amplifiers for each row of the array allows obtaining a voltage Vout1 that is inversely proportional to the measuring resistance Rm (see Equation (1)) while keeping each row reference to ground, thus reducing cross-talk. The output voltage Vout1 is given by:

(5) $$Vout1=-\frac{V_{DRIVE}}{Rm}Rf$$

where Rf is the feedback resistance of the operational amplifier represented in Figure 2. A programmable gain amplifier (PGA) is additionally used in order to obtain a voltage Vout2 suitable to be digitized by the analog-to-digital converter (ADC) at maximum resolution.

### 2.4. Fabrication of the Pressure Sensitive Mat Prototype

**Figure 3:**
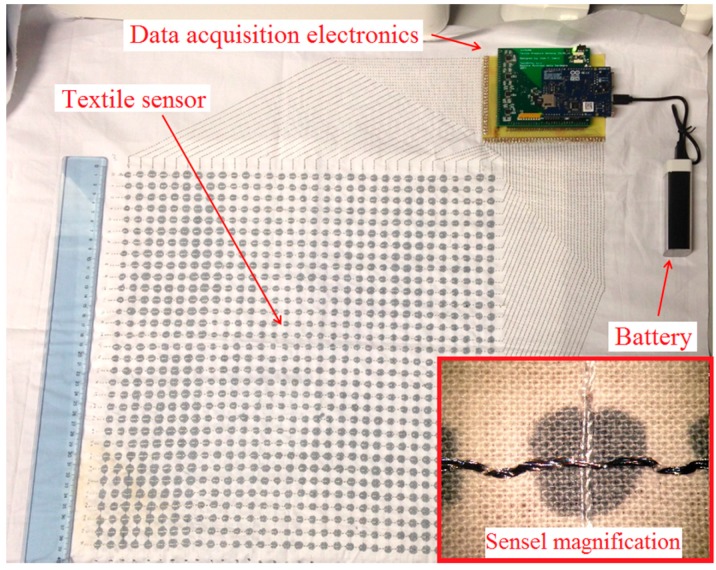
Photograph of the pressure mapping mat prototype without cover layers.

The prototype of pressure sensitive mat consists of an array of 32 × 32 sensing elements fabricated on a single sheet of cotton fabric as shown in Figure 3. The active area is 39 cm × 39 cm (12 mm sensel pitch) which is enough to accommodate an average person standing with their feet a shoulders breadth apart. The sewing paths were designed using CAD software and then converted to stitches and embroidery specifications. Sewing was done automatically by a large-area industrial embroidering machine from Tajima (Kasugai, Japan). In order to connect the conductive sewing paths with the data acquisition electronics, we designed a special printed circuit board (PCB) with large pads close to the board edge that allows sewing and fixing it directly to the fabric. Electric contact between silver-coated threads and PCB copper pads is ensured by applying a conductive epoxy. The treatment of crossing points with PEDOT:PSS for creating the sensing elements was done manually using a small cotton stamp.

**Figure 4:**
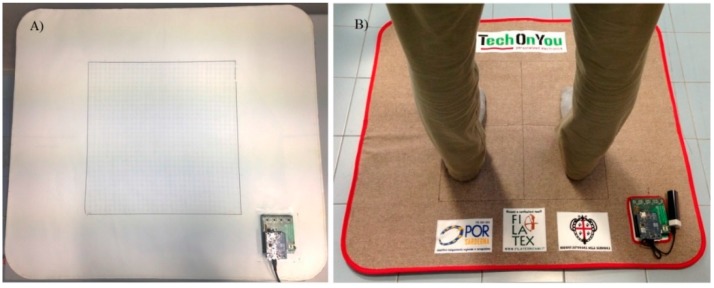
Photograph of the pressure mapping mat prototype with (**A**) impermeable cover only and (**B**) colored cotton cover.

Since water and air moisture affect the conductivity of PEDOT:PSS [22], sensors are covered with a polyvinyl chloride (PVC)-coated polyester fabric which provides impermeability and mechanical protection (see Figure 4A). An additional covering of colored cotton fabric is also added for providing an aesthetic finish and a soft/comfortable interface (see Figure 4B).

The electronic components of the prototype are distributed in two PCBs stacked on top of the other. The lower one contains the analog components and the digital-to-analog and analog-to-digital converters and the top one contains the microcontroller and communication circuitry (see Appendix A for details). This top board is a commercial Arduino Yùn board that includes, in addition to the microcontroller, a system-on-a-chip (SoC) with local area network (LAN) connectivity and an SD card for data recording [23]. The SoC runs with a Linux-based operating system that can be interfaced and programmed through a standard Wi-Fi connection. The device is powered by a Li-ion battery.

**Figure 5:**
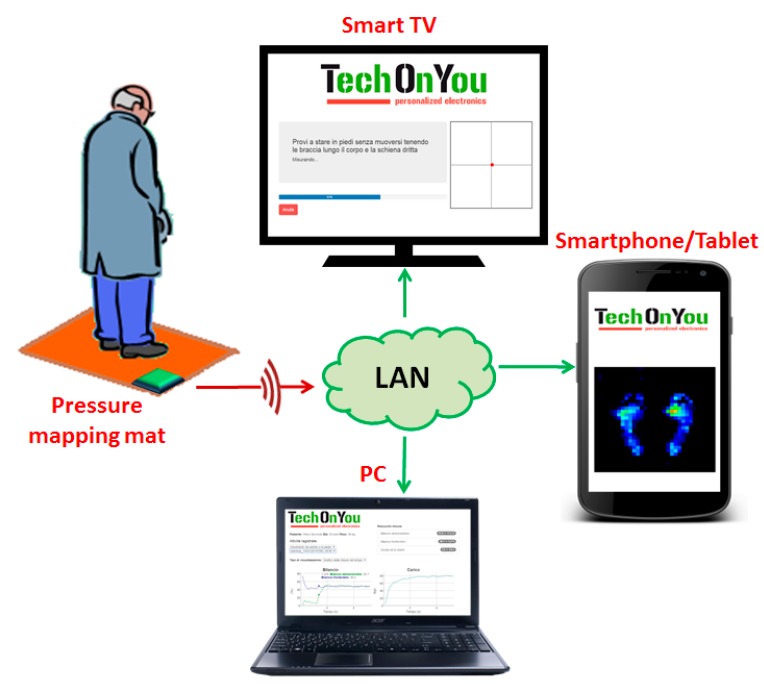
Scheme illustrating the relation between the pressure mapping mat and the user interfaces running on LAN-connected devices as web applications.

In order to optimize the acquisition speed, data was codified with 8-bit resolution which allows processing and storing a single sensel reading in less than 122 μs, thus obtaining a sampling frequency for the entire 32 × 32 matrix of 8.1 Hz. Data acquisition and sensor equalization logic are programmed on the microcontroller firmware using the Arduino development environment. Data recording and sensor calibration is programmed in JavaScript and runs on the SoC under the Node.js environment. A user interface for system configuration and data visualization was also developed and is composed of different web applications written in JavaScript/HTML 5 that can run on the browser of any LAN-connected device (see Figure 5).

### 2.5. Characterization, Equalization, and Calibration

Single sensel measurements were done by using a motorized test stand equipped with a digital force gauge model Imada ZP-50N (Imada CO., LTD., Toyohashi, Japan). Sensel resistance was measured with a Source Meter 2336/2612 (Keithley Instruments, Solon, OH, USA). For the measurements with the mat prototype, we use a homemade setup composed of a manual hydraulic press and an electronic weighing scale. Different forces were applied with a soft testing plate with a fixed area of 81 cm^2^ and pressure was calculated as *Pressure = Force/Area*.

Due to the variability of the sensel-to-sensel response, we implemented an equalization routine that consists on applying a predefined pressure (*i.e.*, 180 kPa) to all sensels and adjusting the value of the voltage $V_{DRIVE}$ required to obtain a predefined output (*i.e.*, 25% of the full digital output). Since we could only apply pressures to a maximum area of 81 cm^2^ at a time, several local equalizations were performed in order to cover the entire sensor. The different values of $V_{DRIVE}$ were stored inside the microcontroller memory and are recalled by the firmware before each reading.

After the equalization, a calibration procedure was still needed to transform the digital data to pressure readings. For this, we applied four different pressures (1 kPa, 60 kPa, 120 kPa, and 180 kPa) to a representative area of the device and calculated the average the response of the involved sensels. Due to limitations of the available instrumentation (the hydraulic press setup), it was not possible to apply higher pressures or to include the whole active area of the device. Given that the resistance of a sensel can be expressed as a rational function of the applied pressure (Equation (4)), and that the measuring voltage is also a rational function of the sensel resistance (Equation (5)) a natural fit the resultant data resulted with a rational function of the form:

(6) $$Y_{fit}=\frac{aP+b}{P+c}$$

where $Y_{fit}$ is the curve that fitted the average digital data best, P is the applied pressure and a, b, and c are the calibration constants resulting from the curve fitting algorithm. Any subsequent measure was calculated using the following expression:

(7) $$P_{cal}=\frac{b-Yc}{Y-a}$$

where Y is the acquired digital value.

### 2.6. Validation

The validation of the proposed mat was carried out by direct comparison with a 70 cm × 40 cm commercial pressure platform model Zebris FDM-S (Zebris Medical GmbH, Allgäu, Germany) which is commonly employed in plantar pressure distribution analysis of healthy and pathological subjects [24,25]. This device is composed by 2560 capacitive sensing elements arranged in a 64 × 40 matrix and is able to acquire pressure data at variable frequencies between 50 Hz (static standing trials) and 100 Hz (walking).

Three healthy volunteers were enrolled in the study to evaluate both static and dynamic performance. Our new mat was superposed to the commercial platform and the following trials were performed:

1. Bipedal static standing. In this test, a subject was asked to stand as still as possible for 30 s in a comfortable position having the feet parallel and placed at a distance approximately correspondent to the shoulder width. Three trials were performed for each subject.
2. Unipedal static standing. Similar to (1) but supporting the body with one limb only having the other suspended at medial malleolus height. Two trials were performed for each subject (one for each foot).
3. Walking (dynamic). The subject walked onto the mat/platform surface taking care to hit the devices with a single foot. The acquisition area of the mat was limited to 8 × 21 sensels in order to increase the acquisition speed to 20 Hz. Three trials were performed for each subject (with the same foot).

The raw data (exported as ASCII files in both cases) were post-processed with a custom routine (developed under Matlab environment) to calculate the following parameters for each foot:

- Contact areas in the forefoot, midfoot, and rearfoot (expressed in mm^2^). Foot segmentation was done according to the procedure proposed by Cavanagh and Rodgers [26]. Contact areas were calculated by summing the area covered for non-zero sensels in each foot zone.
- Mean and peak contact pressure in the forefoot, midfoot, and rearfoot (in kPa).

For static trials, the parameters were calculated from the mean pressure map obtained by averaging all the frames recorded during the 30 s trial. For dynamic trials, the parameters were calculated from the maximum pressure map obtained by extracting the maximum value of each sensel during the entire trial. A total of 33 foot pressure maps were analyzed (18 from bipedal trials, six from unipedal trials, and nine from walking trials). Relative percentage differences (RPD) were calculated taking the measures obtained with the commercial platform as reference in this way:

(8) $$RPD=\frac{Mat_{parameter}-Platform_{parameter}}{Platform_{parameter}}\times 100$$

## 3. Results

**Figure 6:**
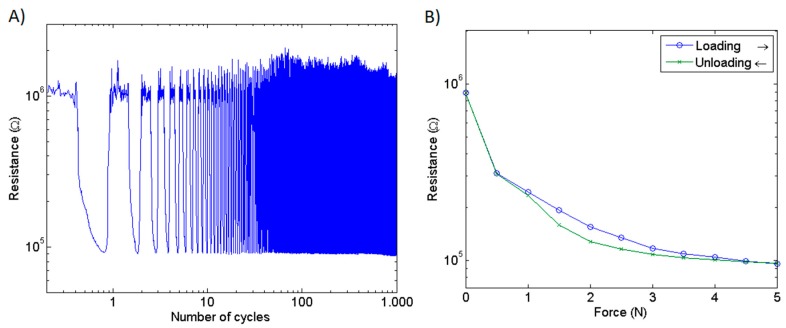
(**A**) Response of a single sensel to a 5 N load over 1000 cycles; and (**B**) typical loading-unloading curve of a single sensel.

The resistive response of a single pressure-sensing element to a load of 5 N over 1000 cycles is shown in Figure 6A. Though the zero load resistance shows some variability, the response to the applied load is stable. In Figure 6B a typical loading-unloading curve is shown.

**Figure 7:**
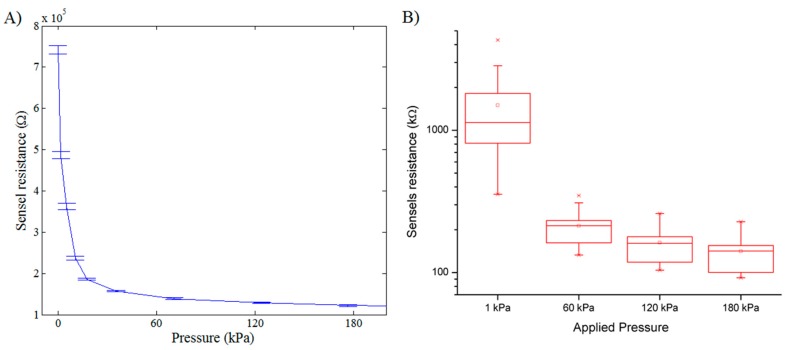
(**A**) Typical response of a single sensel of the mat prototype to the applied pressure. Error bars represent the standard deviation for three independent measures; and (**B**) boxplot that summarizes the response of 16 different sensels of the mat prototype.

**Figure 8:**
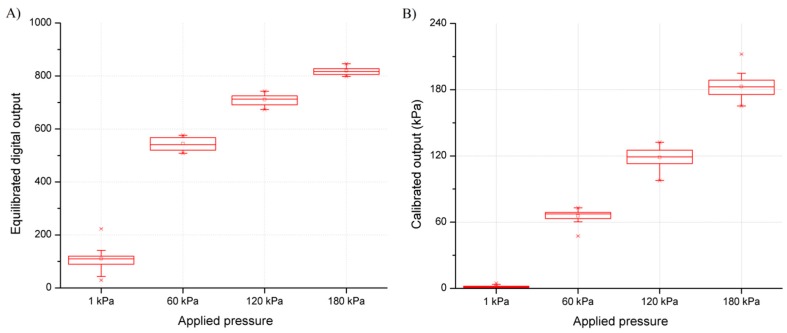
Boxplot of the equilibrated digital readout (**A**) and calibrated pressure output of 16 different sensels of the mat prototype (**B**).

Single sensel and sensel-to-sensel variability of the mat prototype is illustrated in Figure 7A,B, respectively. It can be noted that, though the response of a single sensel is reproducible, sensel-to-sensel variability can be quite high. This variability is due to a variety of reasons: for instance, the contact area between the upper and the lower yarns and the treated portion of the fabric is presumably not exactly the same for each sensor. Additionally, the amount of polymer adsorbed by the fabric depends on the fabric material and is not exactly reproducible in a manual deposition procedure. By automatically depositing the conductive compound, it should be possible to limit this source of variability, even if the textile structure of the fabric does not ensure a perfectly homogeneous density of the textile material in each sensor site. However, this variability is substantially reduced after applying the equalization procedure as observed from the resultant digital readout (Figure 8A). Calibrated outputs of the same sensels are shown in Figure 8B.

**Figure 9:**
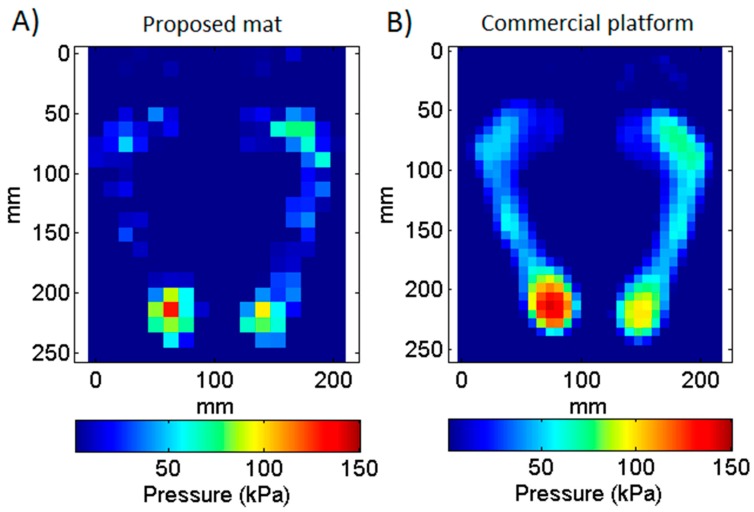
Comparison between two average plantar pressure maps acquired simultaneously with the proposed mat (**A**) and a commercial platform (**B**) during a bipedal standing trial. Maps represent the average pressure during a 30 s acquisition.

**Figure 10:**
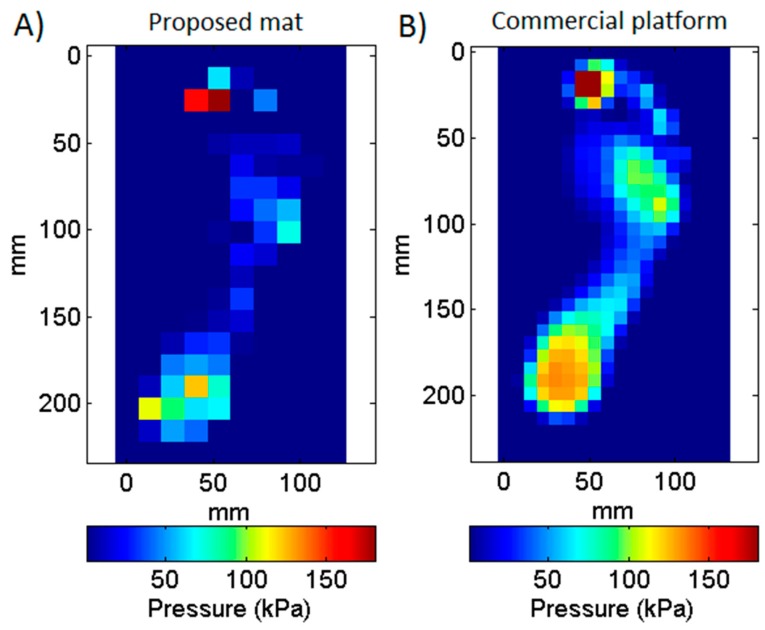
Comparison between two average plantar pressure maps acquired simultaneously with the proposed mat (**A**) and the commercial platform (**B**) during a unipedal standing trial. Maps represent the average pressure during a 30 s acquisition.

**Figure 11:**
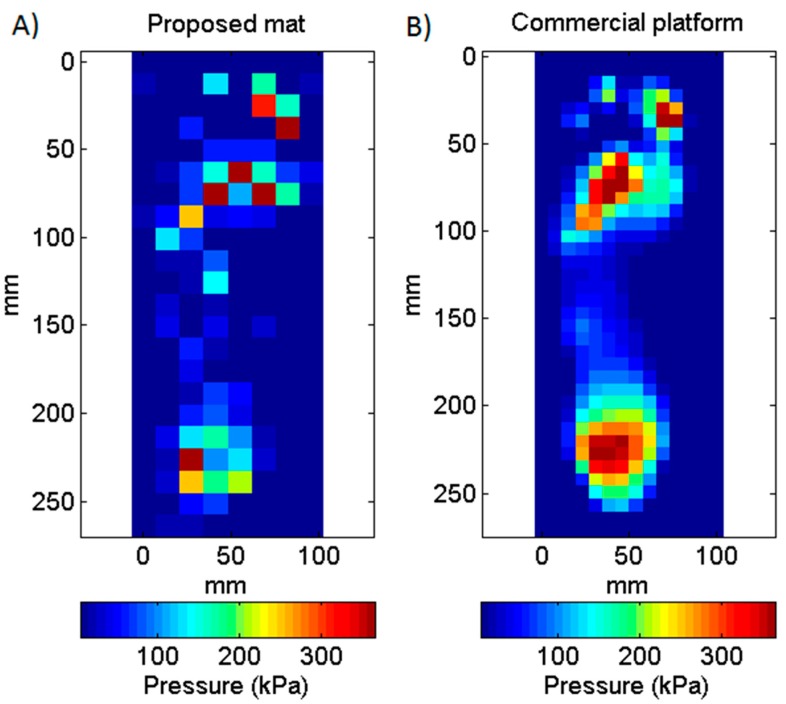
Comparison between two maximum plantar pressure maps obtained with the proposed mat (**A**) and the commercial platform (**B**) during a walking trial. Maps represent the maximum pressure recorded during a single step.

Figure 9 and Figure 10 show examples of average plantar pressure maps from simultaneous recordings made with the proposed mat and a commercial platform during the bipedal and unipedal static trials, respectively (from different subjects). Similarly, Figure 11 show an example of maximum-pressure maps obtained during a walking (dynamic) trial. All maps were randomly selected from all recordings for illustrating a typical case for each type of trial. Pressure maps are scaled to illustrate real device dimensions and are compatible with existing methods of analysis. We did not apply any data transformation or sharpness enhancement algorithm.

Pressure values obtained with the mat show an overall agreement with those obtained with the platform even though a sensel-by-sensel inspection reveal some non-negligible differences. Table 1 allows making a quantitative comparison of this by showing the parameters calculated for each foot zone from the pressure maps shown on Figure 9, Figure 10 and Figure 11 (only the right foot of Figure 9 was considered on Table 1). It can be noted that that contact areas, mean pressures, and peak pressures calculated with the mat were numerically lower than the same parameters calculated with the platform, with the largest differences being observed in the midfoot, which is the region characterized by the lower pressure values.

**Figure 12:**
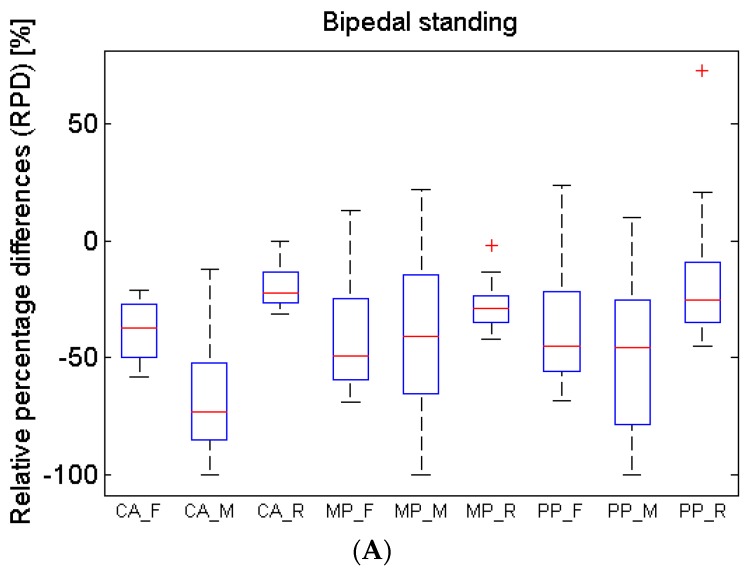

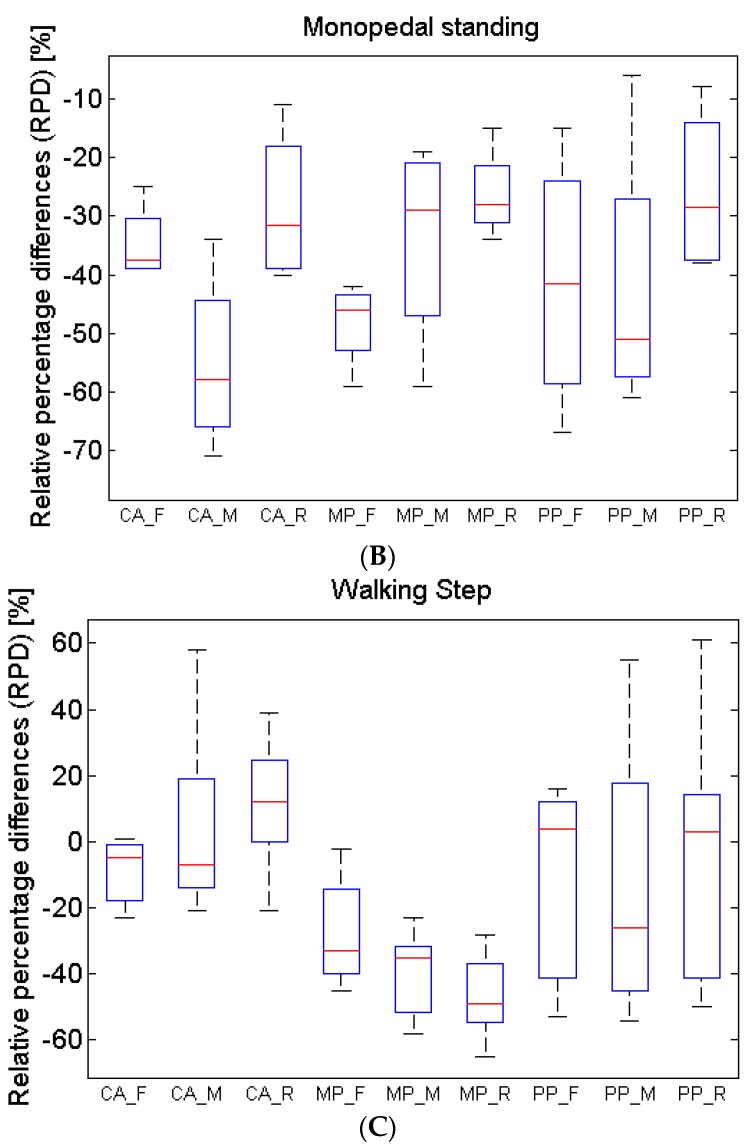
Boxplot of the relative percentage differences (RPD) obtained with each group of trials: bipedal standing (**A**), monopedal standing (**B**), and walking step (**C**). Individual boxplots are identified as CA-F (contact area of **forefoot**), CA-M (contact area of **midfoot**), CA-R (contact area of **rearfoot**), MP-F (mean pressure on **forefoot**), MP-M (mean pressure on **midfoot**), MP-R (mean pressure on **rearfoot**), PP-F (peak pressure on **forefoot**), PP-M (peak pressure on **midfoot**), and PP-M (peak pressure on **rearfoot**).

Figure 12 gives a summary of relative percentage differences (RPD) obtained with all the static and dynamic trials performed. Data from bipedal and unipedal trials is shown in Figure 12A,B respectively, whereas data from walking trials is shown on Figure 12C. As already observed in the sample data of Table 1, the RPD along all trials also show that differences are higher and more spread for midfoot and lower for rearfoot parameters. In general, the RPD for mean pressures are similar in static and dynamic conditions, whereas the RPD averages for peak pressures are lower for the dynamic (walking) measurements. The RPD averages for the contact area are much lower in dynamic measurements.

## 4. Discussion

We have presented a textile pressure sensor with a very sensitive and stable resistive response. In contrast to capacitive textile sensors like those presented by Meyer *et al.* [11] or by Lee *et al.* [14], our sensor can be used with relatively simpler electronics at high-speed, is very thin (less than 1mm thick) and flexible, and uses a minimum amount of expensive materials. Such features are advantageous for large-area or wearable applications that require a high number of sensing elements while maintaining a low cost.

The main part of our sensor is fabricated by sewing and stamping a single layer of a conventional cotton fabric. While it still needs additional cover layers for providing moisture and mechanical protection, such layers are made of conventional low-cost materials and do not participate in the transduction process. Differently than what was reported by Shimojo *et al.* [21], our approach, based on the point-like pressure sensors, avoids cross-talk effects and, in addition, is based on standard, easy-to-sew conductive yarns.

Our sensor is based on the conductive polymer PEDOT:PSS which has known piezoresistive properties that make it suitable for making force-sensitive sensors [27,28]. We are aware, however, that it also has thermoelectric [29] and humidity-sensitive [22] properties. Preliminary experiments (see Supplementary Figure S1) have confirmed that the impermeable cover effectively blocks the loss of conductivity associated with air moisture exposure. Although a precise characterization of the temperature response of our prototype has to be done, preliminary data suggest that temperature sensitivity of our sensor is negligible (see Supplementary Figure S2) given that the target application here implies an indoor environment where temperature variations (presumably very few degrees celsius) arise mainly from the interactions with the human body through several layers of fabric.

The quantity and cost of the materials used in the proposed approach are relatively low when compared with existing solutions for measuring pressure distribution with flexible substrates [8,9,11,12,13,14,15,16]. In terms of fabrication time and human effort, it took us just a few minutes to sew the conductive yarns with the automatic embroidering machine and the stamping of the conductive polymer spots, although done by hand by a single operator, took only two hours to complete. It is also possible to envision different automatic techniques for depositing the conductive polymer, such as screen printing, digital printing, *etc.* Therefore, it is very likely that our approach also implies lower fabrication costs when compared with other textile or fiber-based pressure sensors [11,12,13,14,15,16] if scaled to the same area as ours. Only the plastic film-based devices, like those presented by Tan *et al.* [8] and Papakostas *et al.* [9], may be fabricated with a faster and cheaper procedure since those are based on already industrialized sensors probably based on screen printing methods. However, achieving the same kind of performance on a fully textile platform, rather than on a plastic sheet, paves the way to a variety of new different applications, besides that shown here (*i.e.*, the pressure sensitive mat). In fact, fiber-based materials offer superior comfort, flexibility and mechanical robustness [30]. Those are properties that, for certain kinds of applications (for instance all those based on the contact with human body), are much more interesting than those based on plastics films [30].

The possibility of using electronic embroidering machines allows us to control the features of our sensor with sub-millimeter resolution. The spatial resolution of our technology is, nevertheless, limited by the quality of the conductive yarn and not by the embroidering machines. This is because yarns are made of twisted fibers that can break and stick out during sewing, giving the yarn a hairy aspect. The “hairs” of the conductive yarns can be several millimeters long and produce short circuits between stitching lines that are supposed to be isolated. Given the 12 mm separation between the sensels of our mat prototype, we only observed such short-circuits in less than 2% of the sensels. By manually eliminating these hairs, we can easily go to a sensel separation as low as 7 mm; reasonably, this technology is not suitable for sensors with less than 5 mm of sensel separation.

Since both conductive yarns and substrate fabric are irregular at the microscopic scale, each sensel may absorb the conductive polymer solution in a different way and may have slightly different mechanical properties. Those may be the reasons why there is a high degree of variability in the resistive response between different sensels (see Figure 6B). By carefully selecting a different type of fabric it might be possible to reduce this variability.

Another source of variability is given by the process of manually stamping the PEDOT:PSS on the cotton fabric which produces differences in the size of the treated area between individual sensels. However, since the surface resistivity of the treated fabric is much bigger than the resistivity of the conductive yarns (~10 kOhm/sq *versus* ~5 Ohm/cm), the current in a sensel flows mainly along the shortest path (*i.e.*, the intersection point between the top and bottom conductive yarns). Therefore, it is plausible that variations of the treated area size affect much less the device performance than variations of the geometry and properties of the involved materials at the intersection itself. Most of the variations of the treated area can be easily reduced by automatizing the stamping process.

In any case, the equalization procedure allows us to reduce most of the variability (see Figure 8A). Variability may be further improved by measuring the sensor response with instrumentation of higher quality.

The plantar pressure parameters calculated from the calibrated output of our mat prototype are in line with what has been observed for normal feet in studies reported in the literature [31]. However, the data simultaneously acquired with the commercial platform show that most of the parameters are actually rather underestimated. We think that this is mainly a calibration problem that can be solved by both calculating a different set of calibration parameters for each sensel, and using a calibration setup that allows applying more accurate and homogeneous pressure values over the entire sensor. Since our textile sensor is not completely flat, the last is best achieved by using a setup entirely based on pressure (for instance, a pneumatic setup based on a caged rubber bladder) and not on the force distributed by a solid, like the one we used.

It is important to note that some of the discrepancies observed in the simultaneous measurements were due to the noise induced by the platform circuitry into the textile sensors. This is more evident on the maximum pressure map of Figure 11 (because it is the result of non-averaged data) where it is possible to observe some active sensels outside the actual footprint.

Although the proposed technology may need more development in order to achieve clinically-accurate plantar pressure measurements, results are encouraging and we believe that its applicability for home monitoring and fitness assistance is very promising. In our vision, a pressure mapping mat can be easily positioned in front of a couch or next to the bed of the user in order to monitor the plantar pressure and balance parameters during the sit-to-stand movement which is one of the indicators of the physical condition and falling risk in elderly people [32]. Moreover, this kind of textile technology could be used in all those sensing tasks where flexibility and comfort are desired. For instance, mattresses and cushions for anti-decubitus applications could be another interesting application to explore.

## 5. Conclusions

A pressure mapping mat based on a very thin textile sensor was developed. Since our sensor is made of a single layer of standard cotton fabric with few stitching lines of silver-coated yarns and small pressure sensitive spots treated with PEDOT:PSS, it can be easily fabricated for covering large areas while relying on low-cost processes. The functionality of the device was demonstrated by comparing its performance with a commercial pressure platform considered as the gold standard for biomechanical evaluation of patients; a preliminary validation for measuring plantar pressure parameters was successfully performed. Our results indicate that it is possible to use this technology for obtaining stable and consistent estimations of plantar pressures or force distribution.

## Figures and Tables

**Table 1:** Example of parameters and relative errors for a single trial of bipedal standing, unipedal standing, and walking. Data was extracted from the pressure maps shown in Figure 9, Figure 10 and Figure 11. Parameters were calculated for each foot zone: Forefoot (F), midfoot (M), and rearfoot (R). Platform values are taken as reference for calculating relative percentage differences (RPD).

		Bipedal Standing (Right Foot Figure 9)			Unipedal Standing (Foot Figure 10)			Walking Step (Foot Figure 11)		
		Mat	Platform	RPD	Mat	Platform	RPD	Mat	Platform	RPD
**Contact area [mm^2^]**	**F**	3168	4129	−23%	2448	4027	−39%	4752	4996	−5%
	**M**	1584	2447	−35%	1152	2957	−61%	3024	3314	−9%
	**R**	2016	2804	−28%	2592	2906	−11%	4032	3772	7%
**Mean pressure [kPa]**	**F**	27	35	−23%	23	39	−42%	116	118	−2%
	**M**	16	32	−49%	13	32	−59%	29	43	−31%
	**R**	46	51	−9%	51	77	−34%	86	167	−49%
**Peak pressure [kPa]**	**F**	76	74	3%	72	108	−33%	600	530	13%
	**M**	38	60	−37%	32	81	−61%	147	95	55%
	**R**	97	99	−2%	123	134	−8%	400	365	10%

## Supplementary Materials

The following are available online at http://www.mdpi.com/1424-8220/16/3/365/s1. Figure S1: Effect of exposure to air humidity; Figure S2: Effect of temperature while applying a constant pressure.

## Abbreviations

The following abbreviations are used in this manuscript:

PEDOT:PSS	poly(3,4-ethylenedioxythiophene):poly(styrenesulfonate)
LAN	Local Area Network
CAD	Computer Assisted Design
ADC	Analog to Digital Converter
PGA	Programmable Gain Amplifier
PCB	Printed Circuit Board
SoC	System On a Chip
RPD	Relative Percentage Differences

## Appendix A

This is a list of the main electronic components used and its functions (see Figure 2):

- DAC: Digital-to-analog converter (MCP4921—Microchip Technology): Generate the polarization voltage $V_{DRIVE}$ that drives the sensels. Receive SPI commands from the microncontroller.
- Mux1: Analog multiplexer (ADG731—Analog Devices): Directs the polarization voltage to the column (top conductive path) of the sensel to read. Receive SPI commands from the microncontroller.
- Operational amplifiers (MCP6V14—Microchip Technology) in transimpedance configuration: Transform into voltage the current of each row (bottom conductive paths) of sensels.
- Mux2: Analog multiplexer (ADG731—Analog Devices): Selects the output voltage of the row (bottom conductive paths) where the sensel to read is located. Receive SPI commands from the microncontroller.
- PGA: Programable gain amplifier (MCP6S21—Microchip Technology): Amplify the output voltage Vout1 in order to obtain the voltage Vout2 suitable to be digitalized. Amplifica il voltaggio di misura per la sua corretta digitalizzazione. Receive SPI commands from the microncontroller.
- ADC: Digital-to-analog converter (MCP3001—Microchip Technology): Transforms the output voltage Vout2 to a digital data that is transmitted to the microcontroller using SPI communication.
- Microcontroller (Atmel ATmega32u4 on Arduino Yún board): Implements all the data acquisition logic. Communicates with the System-on-a-Chip (SoC) through a TTL serial interface. It’s programmed in C language using Arduino environment.
- SoC: System-on-a-Chip (Atheros AR9331 on Arduino Yún board): Implements the logic for data storing, acquisition management, web-server tasks and user interfaces back-end. Coomunicates with user interface devices using Wi-Fi communication. It’s programmed in Javascript language under Node.js environment.
