# Supplementary material for: Pressure Mapping Mat for Tele-Home Care Applications
Source: Sensors (Basel). 2016 Mar 11;16(3):365. doi: 10.3390/s16030365 (PMC4813940; doi:10.3390/s16030365)
Supplement: Supplementary file 1 [file sensors-16-00365-s001.pdf]

Supplementary Materials: Pressure Mapping Mat for Tele-Home Care Applications

Jose Francisco Saenz-Cogollo, Massimiliano Pau, Beatrice Fraboni and Annalisa Bonfiglio

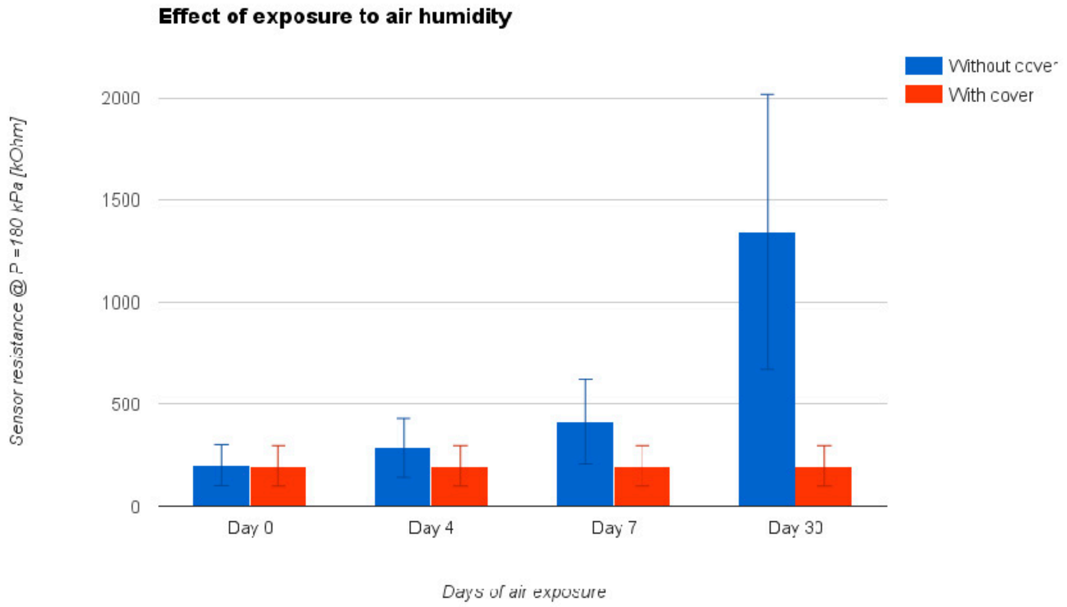

Figure S1. Effect of exposure to air humidity.

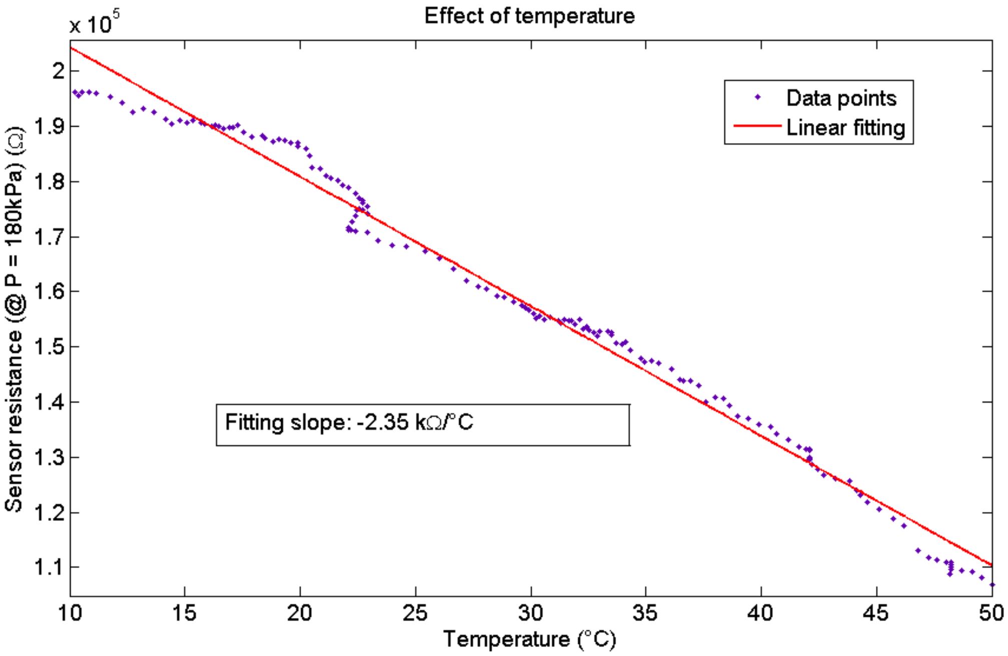

Figure S2. Effect of temperature while applying a constant pressure.
